# Towards Optoelectronic Technology: From Basic Research to Applications

**DOI:** 10.3390/s25072116

**Published:** 2025-03-27

**Authors:** Jacek Wojtas

**Affiliations:** Institute of Optoelectronics, Military University of Technology, 2 Kaliskiego Str., 00-908 Warsaw, Poland; jacek.wojtas@wat.edu.pl

**Keywords:** thermal radiation, infrared radiation, atmospheric infrared transmission, temperature measurement, thermal imaging, scattering, absorption, absorption spectroscopy, technology readiness level, TRL

## Abstract

This editorial is an introduction to the monograph constituting a Special Issue of the *Sensors* journal, which consists of 11 articles of different natures, mainly concerning applications in the latest photonics achievements in ultraviolet, and above all, infrared range of the electromagnetic spectrum. These articles have been grouped into two areas: basic research and application research. Both types of scientific activities have been characterized, emphasizing their complexity and differences, including defining their level of technological advancement. Moreover, regarding application research, the main issues considered fundamental for the research presented there have been identified and described.

## 1. Introduction

Optoelectronic technologies play a key role in a wide range of sophisticated systems and everyday devices. A rapid development of these technologies is driven by the needs of various and increasingly demanding applications, and on the other hand, it is inextricably linked to basic research.

**Figure 1:**
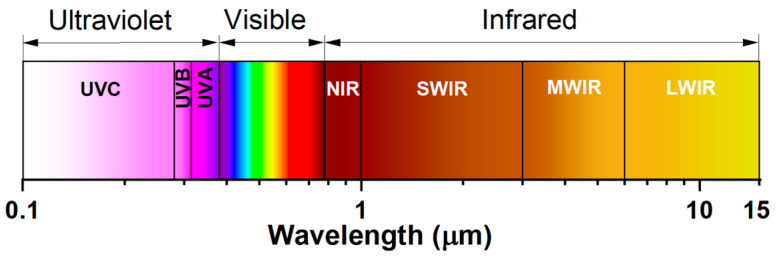
UV-IR wavelength range.

The main purpose of the Special Issue (SI) was to review state-of-the-art photonic knowledge and cutting-edge optoelectronic technologies that can be used in a wide variety of applications. It is a collection of scientific achievements at various levels of technology maturity dedicated to systems operating in the ultraviolet (UV) and infrared range (IR) (Figure 1). It should also be emphasized that these types of technologies are considered dual-use technologies, which strongly favours their rapid development. For example, UV lithography is used to produce the most modern integrated circuits and processors (the size of individual structures in an integrated circuit can reach up to 5 nm). There are a number of everyday, medicine, and military applications such as flame and missile detectors, UV and IR imaging recognition systems, CBRNe detections, contamination and forensic analysis, and communications systems [1,2,3,4,5,6,7].

Taking into account the nature of the presented articles, which fall into two areas of basic and applied research and focus mainly on metrological aspects and new metrological tools, it is worth starting from some general issues. Firstly, scientific activity (science) is a system of knowledge that guarantees objective knowledge of reality. In terms of content, science is a system of duly justified concepts, statements, and hypotheses containing knowledge that is as objective and adequate as possible at a given stage of development of scientific knowledge and practice.

Secondly, activities that constitute scientific knowledge consist of determining and communicating the results obtained from research, and the implementation of these results into practice, as well as the application of scientific research methods, verifying and presenting scientific statements and laws based on true, valid, complete, precise, and orderly scientific facts of the studied subject or area of research. It takes place as a multi-stage, conscious, and purposeful process of diverse cognitive activities.

Thirdly, the scientific activities are regulated by specific principles and research procedures so that the obtained results are complete, precise, reliable, and adequate in relation to the studied reality. Taking this into account, the research procedure must be consistent with scientific methods, ensuring rational selection, ordering, methodological, and substantive correctness of actions and procedures in collecting and considering the obtained data.

Finally, the effect of the research should always be a new and utilitarian result, which may concern the explanation of the problem or the determination and establishment of unknown values and relations between objects, organizations, structures, processes, and other parameters of the studied phenomena.

All articles qualified for publication in the SI are of this nature. What is more, they exhibit the features that have already been cited above, namely, they concern basic and applied research. Basic research aims to theoretically enrich knowledge in a given field or discipline. It aims to discover new truths and relations between phenomena, creating a theoretical foundation and developing new theories. The first three chapters should be included in this type of research:

1. Ultraviolet Photodetectors: From Photocathodes to Low-Dimensional Solids;
2. Theoretical Analysis of GeSn Quantum Dots for Photodetection Applications;
3. Two-Channel Detecting Sensor with Signal Cross-Correlation for FTIR Instruments.

On the other hand, application (applied research) is primarily empirical research. It allows for the formulation of new pragmatic conclusions that can be implemented in practice in order to improve the effectiveness of the action. Regardless of the type of research, the adopted research strategy may force different research approaches and the use of different procedures and research methods in this area. The procedure is understood as a set of directives defining the order of individual stages of research, combinations of methods, and various techniques and research tools. Therefore, the scope of research defined includes the remaining eight chapters:

4. Multi-Spectral Radiation Temperature Measurement: A High-Precision Method Based on Inversion Using an Enhanced Particle Swarm Optimization Algorithm with Multiple Strategies;
5. High-Accuracy Calibration Method of a Thermal Camera Using Two Reference Blackbodies;
6. Efficiency–Accuracy Trade-Off in Light Field Estimation with Cost Volume Construction and Aggregation;
7. Time-Efficient SNR Optimization of WMS-Based Gas Sensor Using a Genetic Algorithm;
8. Multi-Irradiance: A Method for Simultaneous Measurement of the Temperature and Spectral Emissivity of High-Temperature Targets in SWIR;
9. A Diagnostic Method Based on Active Thermography for the Degradation Assessment of Power Plant Boiler Tubes;
10. Polarization Calibration of a Microwave Polarimeter with Near-Infrared Up-Conversion for Optical Correlation and Detection;
11. A Reliable Method of Measuring the Conversion Degrees of Methacrylate Dental Resins.

At this point, it is worth mentioning the Technology Readiness Level (TRL), which defines the path that a given technology has to follow to go from idea to actual product. It is a systematic system that informs how far from implementation a given innovation is. There are many slightly different definitions, but Table 1 presents an interpretation based on the experience in application projects, including the defence sector. It should also be noted that the nine production readiness levels (TRL) correspond to the ten production readiness levels (MRL). MRL supports manufacturers in optimizing risk and allocating resources. The last level (MRL 10) measures aspects of lean practices and continuous improvement for systems in production [8].

To sum up, SI covers the two most attractive areas of optical radiation applications, both commercially and for dual-use or defence systems. The basic issues related to UV and specific types of IR detectors, as well as readout electronics, are described in detail in Chapters 1–3. Therefore, the rest of this article devotes more space to the remaining Chapters 4–11, which deal with infrared. Considering their subject matter, the following subsections of the article describe selected fundamental issues related to thermal radiation, laws, and parameters, as well as temperature measurement, thermography, and infrared absorption.

## 2. Fundamental Aspects of Thermal Radiation

Thermal radiation is the most common form of radiation emission. It is emitted by all objects whose temperatures are greater than absolute zero. An object that completely absorbs incident radiation, regardless of wavelength, is called a perfectly black body (PBB). As a source of radiation, PBB is characterized by the highest possible radiation intensity at a given temperature (and absorption of all incident electromagnetic radiation, regardless of frequency or angle of incidence). In order to describe this effect, Planck put forward the hypothesis in 1900 that an electric harmonic oscillator, which is a model of an elementary source of radiation, can lose energy in the process of radiation emission only in portions, or in quanta, with a value proportional to the frequency *ν* of its natural vibrations [9]:

(1) $$E_{n}=nh\nu ,$$

where the proportionality factor *h* is called Planck’s constant and is equal to 6.626 × 10^−34^ Js, and *n* is a quantum number (*n* = 0, 1, 2…). Assuming that the distribution of oscillators over possible discrete energy states is defined by Boltzmann’s law, then the probability of the oscillators being in a state with energy *nh* at temperature *T* is equal to *p_n_* = *Aexp*(−*nhν*/*kT*), where *A* is a coefficient defined from the normalization condition $\sum_{n}p_{n}=1$. Then, the average energy of the oscillator is

(2) $$M_{e,\lambda }\left(\lambda ,T\right)=\frac{2\pi hc^{2}}{\lambda^{5}}\frac{1}{\exp\left(hc/\lambda kT\right)\;-1}\;[W/(m^{2}\mu m)]$$

This is Planck’s famous formula defining the spectral radiant flux (spectral radiance) emitted by a perfectly black body. This formula defines the spectral distribution of the body’s radiation, which is in very good agreement with the experiment. By integrating this expression over the whole wavelength range, we can calculate the total energy emitted per unit time from a unit area of the blackbody. This gives the total flux emitted by the PBB—called the Stefan–Boltzmann law (*σ_e_* is the Stefan–Boltzmann constant, approximately equal to 5.67 × 10^−12^ Wcm^−2^K^−4^):

(3) $$M_{e}\left(T\right)=\frac{2\pi^{5}k^{4}}{15c^{2}h^{3}}T^{4}=\sigma_{e}T^{4}.$$

By differentiating Formula (2) with respect to *λ* and equating the derivative to zero, we obtain the so-called Wien displacement law. It allows us to determine the wavelength *λ_m_*, at which the spectral existence of radiation *M_e_*_,λ_ reaches a maximum at a specific value of the black body temperature *T*,

(4) $$\lambda_{m}=\frac{2898}{T}\;[\mu m/K].$$

To describe real objects, a spectral emissivity parameter was introduced. It is defined as the ratio of the existence of a real object to the existence of a perfectly black body:

(5) $$\varepsilon \left(\lambda ,T\right)=\frac{M_{e,\lambda }{\left(\lambda ,T\right)}_{Real}}{M_{e,\lambda }{\left(\lambda ,T\right)}_{PBB}}.$$

For a PBB, *ε* = 1 in the entire wavelength range, while for real objects, it is less than unity (*ε* = 1). For example, the total radiation flux emitted by a so-called grey body at temperature *T* is a certain part of *ε* from the corresponding PBB at this temperature in the entire wavelength range:

(6) $$M_{e}^{sz}=\varepsilon \sigma_{e}T^{4}.$$

At this point, it is worth mentioning Kirchhoff’s law, which is an important link to the issues discussed in the chapters mentioned above. It states that the total (integrated) absorption of radiation (α) by a given body is equal to the total emission (α = *ε*). According to the principle of conservation of energy for a body in thermodynamic equilibrium with its environment, the incident flux *Φ_in_* will be equal to the sum of the fluxes: reflected *Φ_ref_*, absorbed *Φ_abs_*, and transmitted *Φ_tr_*

(7) $$\Phi_{in}=\Phi_{ref}+\Phi_{abs}+\Phi_{tr}.$$

By dividing both sides of the equation by *Φ_in_*, one can obtain the relation between the three coefficients: absorption (α), reflection (*r*), and transmission (*t*):

(8) a+r+t=1.

## 3. Basic Application of Infrared Radiation

The first important practical challenge is the remote measurement of the temperature of a distant object. Using an optoelectronic system, we can only measure the radiation flux coming from the object in a wide range of wavelengths or a certain wavelength range ∆*λ*. However, the flux (*Φ*) is a function of both temperature and emissivity. Therefore, information on emissivity is necessary to accurately estimate the temperature with a measurement of the radiation flux. If the emissivity of the object is known, the temperature *T* can be calculated using the Stefan-Boltzmann law, which defines the total energy emitted per unit time from a unit surface of the body. The temperature of the object causing the same radiant exitance as the measured radiant exitance (*M_m_*) is called the radiation temperature *T_R_* [9]:

(9) $$M_{m}=\varepsilon \sigma T^{4}=\sigma T_{R}^{4}$$

(10) $$T=\varepsilon^{-1/4}T_{R}$$

**Figure 2:**
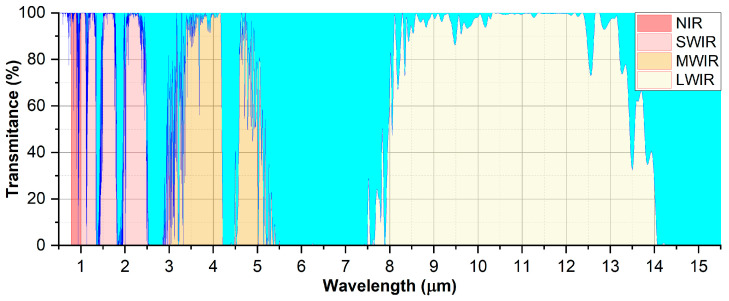
Standard atmospheric infrared transmission with highlighted areas of individual infrared bands determined based on HITRAN database data.

The technique that provides a graphic or visual representation of the temperature conditions on the surface of an object or area is called thermography. Thermography and thermal imaging are rapidly developing fields of science and industry due to the enormous progress made in the technologies of infrared detectors, microsystems, electronic systems, and computer science. In this field, infrared radiation (IR) properties are used, which shows that every object above 0 K emits radiation with energy dependent on the temperature and wavelength. Therefore, it allows for the visualization of the temperature distribution of objects’ surfaces and in some solutions for the accurate measurement of their temperature. Thermography is currently used in scientific research, as well as in many different areas of industry, energy, rescue, construction, medicine, automotive, non-destructive testing, and by the army, police, border guard, and alarm systems. They use different parts of the IR spectrum (Figure 2), which includes four regions [10].

The first region (near-infrared NIR) is limited by wavelengths of 0.78 µm to 1.0 µm and is dominated by reflected solar radiation. For instance, low-light television (L3TV) systems, image intensifiers, and night vision systems operate in this region of the spectrum. The second region is limited by wavelengths from 1.0 µm to 3.0 µm and is called the short-wave infrared region (SWIR). This is a very attractive area for vision systems due to the combination of the advantages of imaging in the visible and infrared ranges. Such cameras record scattered light and the self-radiation (thermal radiation) of objects, which allows seeing despite fog or smog. The third region, the medium-wave infrared (MWIR), is limited by wavelengths from 3.0 µm to 6.0 µm. This range is mainly used to detect and observe objects with higher temperatures (e.g., jet engines). The long-wave infrared region (LWIR) is limited to wavelengths from 6 µm to 15 µm, but because of the high absorbance of water between 6 µm 8 µm, a narrower band, i.e., from 8 µm to 12 µm, is used in practice, mainly for detecting and observing low-temperature objects (e.g., humans).

The main purpose of thermal imaging cameras is to convert infrared radiation into a visible image presented in colours (and less often in greyscale). This image should present a two-dimensional distribution of infrared radiation emitted by the observed object or scene. In the case of a system that allows temperature measurement, the temperature of the individual elements of this object is assigned to a given colour. Thermal imaging cameras use single detectors, detector lines, or detector arrays. In the first two variants, scanning systems are necessary. Scanners made of two mirrors are used for a single detector: for horizontal and vertical scanning. Although scanning systems with a single detector have certain disadvantages related to the need to use complex optical-mechanical units, their undoubted advantage is the radiometric accuracy resulting from the fact that radiation from all image pixels is detected by a single detector [10].

In IR systems that use detector lines, the scene search is performed in one plane, horizontally or vertically. Currently, line scanners are used, among others, for observing the terrain in moving objects (e.g., airplanes or helicopters) or objects moving on a conveyor belt. Advances in the development of infrared detector technology and the development of focal plane arrays (FPA) have contributed to the elimination of scanning systems. Compared to scanning systems, the use of FPAs has enabled a higher number of frames per second with a larger number of pixels. The main advantage of the IR system with detector arrays, apart from the lack of moving mechanical parts and the possibility of using thermal infrared detectors, is that the array covers the entire field of view for the entire duration of the frame. This reduces the bandwidth of each detector, which results in improved SNR. IR detector arrays consist of hundreds of thousands or even more pixels. Unfortunately, due to the imperfections of the technology, the output signal from each pixel, even with uniform illumination of the array, can have a different value. Therefore, calibration of such a device is necessary. In modern cameras, the calibration process is automatic [9,10].

## 4. Some Decisive Atmospheric Factors Affecting Optoelectronic Systems

The Earth’s atmosphere is the most common transmission medium for optical radiation of optoelectronic systems. It is usually a mixture of various substances in the gaseous state and solid particles, with a density dependent on temperature and altitude. They interact with passing photons due to the extinction effect, which is based on the phenomena of scattering and absorption. Scattering can be generally defined as the redirection of radiation from its original direction of propagation, usually due to interactions with molecules and particles. Reflection, refraction, and diffraction are forms of scattering. The relationship between the size of the scattering particles and the wavelength of the radiation is an important factor determining the type of scattering (mathematical model), usually expressed as a dimensionless quantity parameter *x* [11]:

(11) x=2πr/λ

where *r* is the radius of the spherical particle and *λ* is the wavelength. Types of scattering depending on *x*:

(1) *x* << 1: Rayleigh scattering;
(2) *x* ~ 1: Mie scattering;
(3) *x* >> 1: geometric scattering.

In the first case, when the particle size is much smaller than the wavelength of the incident light, the scattering efficiency *Q_scat_* is inversely proportional to λ^4^ and is given by the formula

(12) $$Q_{scat}=\frac{2^{7}\pi^{4}r^{4}}{3\lambda^{4}}{\left|\frac{m^{2}-1}{m^{2}+2}\right|}^{2},$$

where *m* is the complex refractive index. Mie scattering occurs when the particle diameter is approximately the same as the incident wavelength. Then, the mathematical model for isotropic and homogenous spherical particles of any given size is more complicated:

(13) $$Q_{scat}=\frac{\lambda^{2}}{2\pi^{2}r^{2}}\sum_{n=1}^{\infty }\left(2n+1\right)\left({\left|a_{n}\right|}^{2}+{\left|b_{n}\right|}^{2}\right),$$

where *a_n_* and *b_n_* are the Mie coefficients depending on the size parameter and complex refractive index (*m*). In the last one—geometric scattering—particles are much larger than the incident wavelength. The scattering of such large particles can be calculated according to geometrical optics. In practice, the dominant scattering factor is Mie scattering by small particles (dust, water droplets), and only a small part is due to Rayleigh scattering by atoms and molecules [12].

The second type of interaction of photons with matter, which is the absorption of incident radiation, also has a decisive influence on the way optoelectronic systems are designed. Namely, the effect of this interaction is the existence of so-called atmospheric transmission windows. They are defined for wavelength ranges in which optical radiation transmission is possible. In Figure 2, at least eight such windows can be identified, located in the NIR, SWIR, MWIR, and LWIR ranges. The width of these windows depends primarily on the concentration of water vapour (H_2_O) and carbon dioxide (CO_2_), and to a lesser extent on other gases, such as methane (CH_4_) and nitrogen dioxide (N_2_O). Due to the fact that the absorption phenomenon is the subject of several chapters of this monograph, the next section will be devoted to a more detailed description of this phenomenon and the possibilities of its use in applications. To summarize this section, it should be emphasized that the extinction efficiency *Qext* is the sum of the absorption and scattering efficiency coefficients [11]:

*Qext* = *Qabs* + *Qscat*. (14)

## 5. Application of IR Absorption Phenomenon

When the infrared radiation of the appropriate wavelength is incident on the molecule, a change in the molecule’s vibrational and/or rotational energy occurs. The wavelength of the radiation related to the phenomenon corresponds to a single narrow spectral line having a typical width (in air) of the order of hundredths of a nanometre. Because vibrational-rotational states form complex and dense structure energy levels, spectral lines corresponding to transitions between these levels form groups called bands. Generally, compounds have characteristic absorption bands at certain wavelengths. Based on the knowledge of the shape and distribution of these bands, it is possible to identify molecules [12].

Assuming that the molecule is an ideal quantum oscillator and an ideal quantum rotator, an absorption transition may take place between neighbouring states. Because of the anharmonicity of the potential bonding of the molecules, overtone transitions of various orders occur as well, and their corresponding overtone band is also observed.

In practice, it is difficult to predict the structure of the absorption spectrum. Often it is different from the calculated spectrum obtained during measurements, e.g., due to inactive oscillations in the infrared or because of the symmetry of the molecule, which causes the degeneration of certain energy levels. This is because the absorption transition between different oscillation states occurs for the same wavelength.

The single absorption line has the shape of a bell curve with a predetermined width. Under the conditions in which the experiments were carried out, i.e., during temperatures close to room temperature and atmospheric pressure, the most important is the broadening due to the collision of test molecules with other air molecules. It can be minimized by reducing the pressure. A relatively small impact is caused by the Doppler effect resulting from the chaotic motion of the molecules. In contrast, of completely negligible importance is the broadening of the spectrum resulting from the widening of energy levels as described by the Heisenberg uncertainty principle [12].

Spectra with clearly separated lines of the oscillation and rotation can be observed for light particles. In the case of complex polyatomic molecules, usually, the oscillation-rotation structure of the spectra is very complex, and because of broadening, the individual lines overlap and a continuous band is observed.

**Figure 3:**
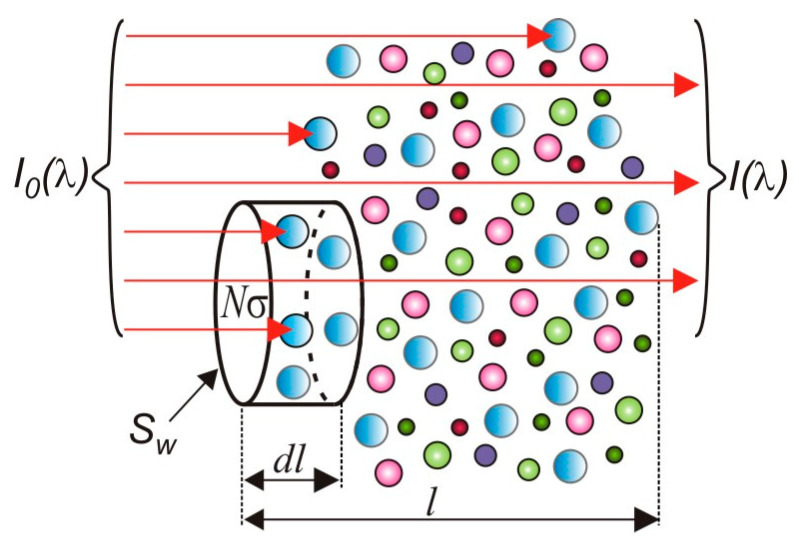
Graphical interpretation of the absorption cross-section.

The absorption of optical radiation is usually characterized by the absorption coefficient *α* describing the weakening of the intensity of radiation passing through the medium. This factor can be defined as the inverse of the film thickness (expressed in m^−1^) when the radiation intensity decreases e-fold. To describe the absorption, the Lambert-Beer law is used [13]. It says that the intensity *I_O_* monochromatic optical radiation of wavelength *λ* after passing through a sample of thickness *l* is reduced to values *I* and in accordance with the equation

(15) $$I(\lambda )=I_{o}(\lambda )\exp(-\sigma (\lambda )\cdot N\cdot l),$$

where *N* is the number of particles per cubic centimetre (concentration), and *σ*(*λ*) is a characteristic constant of the substance, called the absorption cross-section. This parameter is dependent on the wavelength and is expressed in units of cm^2^. In contrast to the absorption coefficient, the absorption cross-section describes the properties of a single atom or molecule under certain conditions (depending on the temperature, pressure, and the type of surrounding medium). It can be defined as a probability of photon absorption of a specific wavelength by a single atom or molecule. Figure 3 shows a graphical interpretation of the absorption cross-section.

Let us assume that the beam of monochromatic radiation with cross-sectional area *S_W_* runs through the absorber. The number of photons absorbed in the elementary layer with a thickness *dl* is proportional to the number of incident photons and the volume *dV* = *dl* · *S_w_*. It is also proportional to the concentration of *N* particles contained in this volume and the absorption cross-section *σ*, which determines the effective surface molecule in the process.

The ratio of the number of photons absorbed ∆*n_f_* to the incident *n_f_* is called a probability of photon absorption, and is proportional to the ratio “particle surface” sectional area *S_W_*

(16) $$\frac{\Delta n_{f}}{n_{f}}=\frac{\sigma }{S_{w}}\cdot dV\cdot N.$$

Between the absorption coefficient *α* (expressed in cm^−1^) and the cross-section for the absorption, the following relationship occurs:

(17) α(λ)=σ(λ)⋅N.

The absorption cross-section can also be expressed by the formula

(18) σ(λ)=S⋅g(λ),

where g(λ) is the normalized absorption line shape function ($\int_{0}^{\infty }g(\lambda )d\lambda =1$) and *S* is the line strength. The line strength, as a parameter independent of the interaction of the molecule with the environment, is often provided in absorption spectra databases, e.g., the HITRAN database.

**Figure 4:**
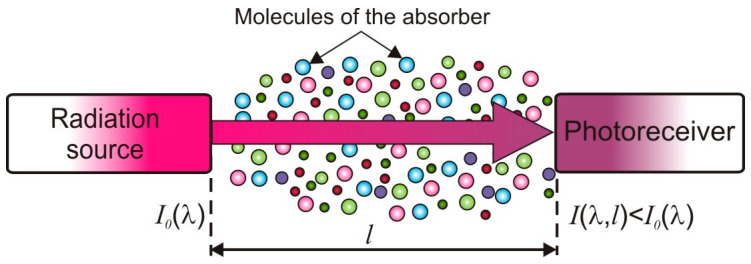
Principle of the absorption method of gas detection.

In absorption spectroscopy, the radiation source may be a lamp, light-emitting diode or laser, the spectrum of which is matched to the absorption bands of the test gas. When an absorber exists between the source and the photoreceiver, the intensity of radiation reaching the photoreceiver is attenuated (Figure 4). On this basis, an absorber concentration can be concluded. The type of absorber can be identified by studying the absorption spectrum.

Based on Equation (1), the detection limit can be expressed in terms of the minimum detectable value of the absorption coefficient (*α_min_*). Then, it is defined by the determination accuracy of the small changes in intensity of laser radiation $\Delta I/I_{0}$, where $\Delta I=I_{0}-I$. Therefore, the noise of the measuring system, fluctuations in the power and in the wavelength of the laser, and the noise of the photoreceiver have a significant influence on the value of *α_min_*. The uncertainty of the method is determined by the uncertainty of the Δ*I* measurement. For very small values of *σ*(*λ*), this uncertainty increases. To obtain a small limit of detection, it is necessary to minimize the various sources of noise. Despite these treatments, in the systems of direct detection (e.g., presented in Figure 2), *α_min_* reaches the value of about 10^−2^–10^−4^ cm^−1^ [14].

A lower detection limit can be achieved by lengthening the optical path of the radiation (*l*), e.g., using multi-pass cells (in Herriot configuration, White, or in astigmatic, etc.). Lengthening the optical path is achieved due to multiple reflections of optical radiation between the mirrors. Multi-pass cells provide an optical path 10–100 times longer (and even more). Thanks to this, it is possible to measure the absorption coefficient in the order of 10^−7^ cm^−1^. A detection limit of 10^−9^ cm^−1^ can be obtained using wavelength modulation (WM) and the phase-sensitive detection procedures realized by lock-in amplifiers. These types of cells are often used in Fourier transform infrared spectroscopy (FTIR) measurements, providing ppm-level sensitivity in gas studies [15,16].

Another solution is to use optical cavities (resonators), which provide optical paths even of several kilometres. This enables measurements of the absorption coefficient of up to 10^−14^ cm^−1^. Such a detection limit is achievable in the cavity ring-down spectroscopy (CRDS) method and in the cavity-enhanced absorption spectroscopy (CEAS) method. CRDS was proposed to determine the reflectance mirrors by J. M. Herbelin in the early 1980s. In the CEAS, which was presented by R. Engeln in 1998, a misaligned coupling of the laser radiation and the optical cavity is used. As in the CRDS, optical cavities use mirrors with very high reflectance (*R*), most often greater than the value of 99.99%. The advantages of this method and the aspects of the design of the sensors, the optical system and the signal processing system, concerning the parameters of the elements available in the market are shown in [12,17].

## 6. Summary

This work is an introduction to the subsequent chapters, which cover the issues mentioned above, relating to two very attractive areas of application of UV and IR optical radiation. Selected phenomena on which the individual achievements presented in the Special Issue are based are characterized; among others, the laws related to thermal radiation and the propagation of radiation in the atmosphere. Thanks to this, the entire book has acquired a more coherent character, addressing issues related to optical radiation detection, various application in infrared systems, and metrological issues.

## Figures and Tables

**Table 1:** Technology readiness level definitions.

Phase	Result	Technology Readiness Level
Scientific research	Knowledge	TRL I—observation and description of basic principles. The beginning of the development of technology is understood as formulated theoretical knowledge that can be verified measurably.
	Concept	TRL II—defining the technology concept related to the future application of technology. Activities are limited to analytical studies (publications) and analyses confirming the assumptions of the application of technology.
		TRL III—proof of concept analytically and experimentally confirming critical functions or characteristics of a technology that includes components that are not yet integrated into or representative of the full technology.
	Components	TRL IV—verification of technology components in a laboratory setting. Basic technology components are integrated ad hoc in the laboratory to confirm that they will work together.
		TRL V—verification of technology components under simulated operating conditions. Basic technology components are integrated with elements simulating real elements and verified in tests reflecting the intended application.
	Lab model, Demonstrator	TRL VI—technology demonstration in near real-world conditions. Testing of a model or technology demonstrator in a laboratory reflecting simulated operating conditions. The use of commercially available components with reduced resistance is still possible.
Development work	Prototype	TRL VII—demonstration of a technology prototype in operational conditions, e.g., on an aircraft, in a vehicle, in an operational IT environment or space.
	End of Technology Development	TRL VIII—confirmation that the technology can be used in its final form and under the conditions expected for it, e.g., within a defence system, to confirm the design assumptions. This level represents the end of the actual development of the technology.
	Application	TRL IX—testing the developed technology in an operational environment. The technology is then applied in its final form and under the expected operating conditions, e.g., in the operational conditions of the mission or the actual operational environment.

## References

[1] Paul T., Roy Choudhury D., Ghosh D., Saha C. (2024). Advancements in optical sensors for explosive materials Identification: A comprehensive review. Results Chem..

[2] Kowalski M.Ł., Pałka N., Młyńczak J., Karol M., Czerwińska E., Życzkowski M., Ciurapiński W., Zawadzki Z., Brawata S. (2021). Detection of Inflatable Boats and People in Thermal Infrared with Deep Learning Methods. Sensors.

[3] Gorajek L., Gontar P., Jabczynski J., Firak J., Stefaniak M., Dabrowski M., Orzanowski T., Trzaskawka P., Sosnowski T., Firmanty K. (2020). Characterization of Absorption Losses and Transient Thermo-Optic Effects in a High-Power Laser System. Photonics.

[4] Gomółka G., Nikodem M. (2024). Gas leak detection by measuring dilution of ambient air with differential optical dispersion spectroscopy of oxygen. Opt. Express.

[5] Drozdowska K., Smulko J., Zieliński A., Kwiatkowski A. (2025). UV light-activated gas mixture sensing by ink-printed WS2 layer. Sens. Actuators B-Chem..

[6] Liszewska M., Bartosewicz B., Budner B., Nasiłowska B., Szala M., Weyher J.L., Dzięcielewski I., Mierczyk Z., Jankiewicz B.J. (2019). Evaluation of selected SERS substrates for trace detection of explosive materials using portable Raman systems. Vib. Spectrosc..

[7] Mikołajczyk J. (2021). A Comparison study of data link with medium-wavelength infrared pulsed and cw quantum cascade lasers. Photonics.

[8] https://learn.forclimatetech.org/what-is-manufacturing-readiness-level-mrl/.

[9] Rogalski A., Bielecki Z. (2022). Detection of Optical Signals.

[10] Rogalski A., Chrzanowski K. (2014). Infrared devices and techniques (revision). Metrol. Meas. Syst..

[11] Signorell R., Reid J.P. (2010). Fundamentals and Applications in Aerosol Spectroscopy.

[12] Demtröder W. (2015). Laser Spectroscopy 2 Experimental Techniques.

[13] Hanson R.K., Spearrin R.M., Goldenstein C.S. (2016). Spectroscopy and Optical Diagnostics for Gases.

[14] Wojtas J., Mikolajczyk J., Nowakowski M., Rutecka B., Medrzycki R., Bielecki Z. (2011). Applying CEAS method to UV, VIS, and IR spectroscopy sensors. Bull. Pol. Acad. Sci..

[15] D’Arco A., Mancini T., Paolozzi M.C., Macis S., Mosesso L., Marcelli A., Petrarca M., Radica F., Tranfo G., Lupi S. (2022). High Sensitivity Monitoring of VOCs in Air through FTIR Spectroscopy Using a Multipass Gas Cell Setup. Sensors.

[16] Kluczynski P., Lindberg Å.M., Axner O. (2001). Characterization of Background Signals in Wavelength-Modulation Spectrometry in Terms of a Fourier Based Theoretical Formalism. Appl. Opt..

[17] Wojtas J., Bielecki Z. (2008). Signal processing system in cavity enhanced spectroscopy. Opto-Electron. Rev..

